# Antidiabetic, antihyperlipidemic and antioxidant properties of ethanol extract of *Grewia asiatica* Linn. bark in alloxan-induced diabetic rats

**DOI:** 10.1186/s12906-016-1276-9

**Published:** 2016-08-18

**Authors:** Naznin Ara Khatune, Bytul Mokaddesur Rahman, Ranjan Kumar Barman, Mir Imam Ibne Wahed

**Affiliations:** Department of Pharmacy, Faculty of Science, University of Rajshahi, Rajshahi, 6205 Bangladesh

**Keywords:** *Grewia asiatica*, Antidiabetic, Antihyperlipidemic, Antioxidant activity

## Abstract

**Background:**

Inspite of introduction of oral hypoglycemic agents, diabetes and its related complications remains to be a major clinical problem. The aim of this study was to investigate the antidiabetic, antihyperlipidemic and antioxidant activities of *Grewia asiatica* (Linn) stem bark in alloxan induced diabetic rats.

**Methods:**

Diabetes was induced by a single dose of intraperitoneal injection of alloxan (110 mg/kg) in Norwegian Long Evans rats. Ethanol extract of barks from *Grewia asiatica* (GAE 200 and 400 mg/kg) and metformin (150 mg/kg) were orally administered once daily for 15 days. Blood glucose levels and body weights of rats were measured on 0, 5, 10 and 15 days of oral treatment. At the end of the experiment the rats were sacrificed and blood sample were collected for the measurement of total cholesterol (TC), triglycerides (TG), very low density lipoproteins (VLDL), low density lipoproteins (LDL), high density lipoproteins (HDL), SGOT and CK-MB. Analysis of liver glycogen content and histopathlogy of pancreas were carried out. In vitro DPPH free radical scavenging activity, total phenolic and total flavonoid content of GAE were also determined.

**Results:**

After 15 days of oral administration of GAE at doses of 200 and 400 mg/kg increased survival rate and showed a significant attenuation in blood glucose and lipid profile in diabetic rats. Oral ingestion of GAE significantly reduced the SGOT and CK-MB levels and restored liver glycogen content when compared to diabetic control. The effects of GAE on SGOT, CK-MB and liver glycogen content were dose-dependent. The diabetic rats treated with GAE showed significant improvement in normal cellular population size of islets. Phytochemical screening of GAE revealed the presence of flavonoid, steroid, tannin and phenolic compounds. Total phenolic content was 44.65 ± 3.17 mg of gallic acid equivalent per gm of GAE extract and the total flavonoid content was 39.11 ± 4.65 mg of quercetin equivalent per gm of GAE extract. In DPPH scavenging assay, IC_50_ values of GAE and ascorbic acid were found 76.45 and 12.50 μg/ml, respectively.

**Conclusion:**

We demonstrated that ethanol extract of barks from *G. asiatica* possess glucose, lipid lowering efficacy, restored liver glycogen and protects pancreas from oxidative damage in alloxan-induced diabetic rats. Thus, the results of the present study provide a scientific rationale for the use of *G. asiatica* in the management of diabetes and its related complications.

## Background

Diabetes mellitus is a metabolic disorder characterized by loss of glucose homeostasis occurring due to defects in insulin secretion or insulin action resulting from impaired metabolism of glucose, lipids and other energy yielding fuels such as lipids and proteins [1]. It is a major endocrine disorder affecting nearly 10 % population all over the world [2]. Globally diabetes has shadowed the spread of modern lifestyle and it can be linked to an increase in overweight and sedentary population [3]. Despite the great strides that have been made in the understanding and management of diabetes, the disease and its related complications are increasing at an alarming rate [4].

Patients with diabetes have dyslipidemia and an increased risk of stroke, coronary heart disease, myocardial infarction and peripheral vascular disease [5]. Hyperglycemia, the primary clinical manifestations of diabetes is thought to contribute to diabetic complications by altering vascular cellular metabolism, vascular matrix molecules and circulating lipoproteins [6]. There are also multiple abnormalities of lipoprotein metabolism in very low density lipoprotein (VLDL), low density lipoprotein (LDL) and high density lipoprotein (HDL) in diabetes. It is now well established that hyperlipidemia represents a major risk factor for the premature development of atherosclerosis and its cardiovascular complications [7]. The American Heart Association (AHA) has identified the primary risk factor associated with progression of atherosclerotic lesions as elevated levels of total cholesterol (TC) and triglycerides (TG) in serum [8]. So, diabetes is a multifactorial diseases leading to several complications require a multiple therapeutic approach. Many investigations suggested that the medicinal plants and dietary supplements improves diabetic conditions by lowering lipid and glucose levels and are useful in the management of diabetic complications especially its associated cardiovascular risks [9].

The most commercially available antidiabetic agents are expensive and possess undesirable side effects such as potential for induction of hypoglycemia, weight gain, gastrointestinal disturbances and liver toxicity [10]. In recent years, complementary medicines are gaining popularity worldwide because of their natural origin and less side effects. Over the years, various medicinal plants have been reported to be effective in the management of diabetes mellitus [11]. The hypoglycemic and hypolipidemic effects of some medicinal plants have been evaluated and confirmed in human [12] and animal models [13, 14] and however, many remained to be scientifically established.

*Grewia asiatica* L. (Tiliaceae) is an exotic bush plant, known for its edible ripe fruit which are consumed fresh [15]. The plant is native to the Indian subcontinent and now widely cultivated on a commercial scale in India, Bangladesh, Pakistan, Philippines and other tropical countries [16]. Traditionally, the plant *G. asiatica* widely used for its antidiabetic, antioxidant, antipyretic, analgesic, antibacterial properties [17]. The plant reported to contain glycoside, flavonoids, vitamins A and C, minerals and dietary fiber [18–21]. Earlier studies have shown the free radical scavenging activity and radioprotective efficacy of *G. asiatica* fruit extract in brain [22], liver and blood [23]. *G. asiatica* leaves has been shown to possess hypoglycemic activity in diabetic rats [24]. Parveen et. al investigated the comparative anti-hyperglycemic effects of crude ethanolic extracts of the fruit, stem bark and leaves of *G. asiatica* and their fractions in alloxan-induced hyperglycemic rabbits after acute treatment [25]. So, we have evaluated the antidiabetic, hypolipidemic and antioxidant effects of ethanol extract of stem bark from *G. asiatica* (GAE) in alloxan induced diabetic rats after 15 days of oral administration.

## Methods

### Drug and chemicals

The standard drug, Metformin HCl was the generous gift sample obtained from Square Pharmaceuticals Ltd., Pabna, Bangladesh. Alloxan monohydrate was purchased from Sigma-Aldrich Co. Germany. All other chemical and solvent used were of analytical grade.

### Plant material

The fresh stem barks of the plant *G. asiatica* were collected from botanical garden of Rajshahi University, Rajshahi, during the month of June-July in 2011. The authenticity of the plant was confirmed and a voucher specimen collection # 29, dated 06/30/2011 was kept in the Herbarium, Department of Botany, University of Rajshahi, Bangladesh.

### Preparation of plant extracts

The collected stem barks were washed, chopped into small pieces and sun dried for several days. The dried stem bark grinded to coarse powder after drying in an oven at below 50 °C. The powdered plant materials were soaked with 3 L of rectified spirit (96 % ethanol) for 7–10 days with occasional shaking and stirring. The extracts thus obtained were successively filtered through cotton and filter paper (Whatman Filter Paper No. 1). The filtrate was defatted with petroleum ether for several times. The defatted liquor was concentrated using a rotary evaporator at 40–45 °C under reduced pressure and finally, the extract kept into a desiccator to obtain a solid mass (yield 30.0 g; 3.0 %).

### Phytochemical screening tests

Detection of phytoconstituents has been performed by the standard methods [26, 27].

### Animals

Nine-weeks-old Norwegian Long Evans rats (150–180 g) purchased from ICDDRB, Dhaka, Bangladesh were housed in cages in an air controlled room under light and dark cycle conditions. Rats were allowed to access standard rodent chow and water *ad libitum*. Throughout the study the animals were cared in accordance with the guidelines of our institution. The experimental protocol was approved by Institutional Animal, Medical Ethics, Biosafety and Biosecurity Committee (IAMEBBC) at the Institute of Biological Sciences, University of Rajshahi, Bangladesh.

### Acute toxicity study

The acute oral toxicity study was carried out according to OECD guidelines. After administration of a fixed dose of 2000 mg/kg of extract, animals were individually observed for any change in autonomic or behavioral response for first 2 h, periodically during first 24 h and daily thereafter, for a total of 14 days [28].

### Induction of experimental diabetes

After fasting 16 h, diabetes was induced into rats by a single intra-peritoneal (i.p.) injection of alloxan monohydrade (110 mg/kg body weight) following base-line glucose estimations. After 96 h blood glucose levels were measured by glucometer using blood sample obtained from tail-vein of rat. Rats with blood sugar level higher than 11.5 mmol/L were considered for the treatment protocol [29].

### Experimental protocol

Twenty diabetic rats were divided into four groups and each group comprised of five animals. The standard drugs and/or extracts were suspended in vehicle (0.5 % methyl cellulose, MC) and administered orally in rats by gastric tube for 15 days. Age-matched healthy rats were used as normal control.

1. Normal Control (Group NC, 0.5 % MC, *n* = 5)
2. Diabetic Control (Group DC, 0.5 % MC, *n* = 5)
3. Diabetic + Standard Drug (Group DS, Metformin, 150 mg/kg, *n* = 5)
4. Diabetic + Extract (Group GAE200, 200 mg/kg, *n* = 5)
5. Diabetic + Extract (Group GAE400, 400 mg/kg, *n* = 5)

### Oral glucose tolerance test (OGTT)

Blood glucose level of rats were measured after fasting over-night. After 1 h of feeding of extracts and/drugs rats received glucose solutions (2 g/kg). Blood samples from each rat were withdrawn from the tail-vein at 0 min, before and after 30, 60 and 120 min of glucose loading. Plasma glucose levels were estimated using glucose oxidase-peroxidase method [30].

### Time course of changes in blood glucose levels

The blood glucose levels of rats were measured on day 0, before initiation and on 5, 10 and 15 days during the course of treatment. Blood samples were drawn from the tail-vein of rats and blood glucose levels were measured [30].

### Measurements of body weights and organ weights

The body weights of rats were measured before the initiation and after 15 days of oral treatment. At the end of experiment, the rats were anesthetized, chest opened, blood samples were withdrawn directly from aorta and poured into blood collecting tube. The blood samples were centrifuged at 4000 rpm for 10 min and the plasma samples thus, obtained were freeze up at −40 °C until further use. Heart, liver and pancreases were removed and cleaned of the surrounding tissues. The organ weights were measured immediately and the organ weight to body weight ratios were calculated. Samples of pancreas were stored in 10 % formalin for histopathological examination.

### Analysis of lipid profile

Plasma triglycerides (TG), total cholesterol (TC) and high density lipoprotein (HDL) concentrations were analyzed by spectrophotometer (Shimadzu 1200, Japan) using commercial kits (Human, Germany). The low density lipoprotein (LDL) and very low density lipoprotein (LDL) levels were determined by the formula, VLDL=TG/5, LDL=TC-(HDL+VLDL) [31]. The ratios of LDL to HDL cholesterol were calculated.

### Estimation of liver glycogen, SGOT and CK-MB levels

Estimation of CK-MB was done by immuno-inhibition method as described by the manufacturer protocol [32]. The liver enzyme, serum glutamate oxaloacetate transaminase (SGOT) was determined using commercial kits (Human, Germany) [33, 34]. The liver glycogen content was determined according to the method described by Tarnoky K. et al., [35]. Briefly, it utilizes the o-toluidine-glucose coupling reaction for the estimation of glycogen after extraction with trichloroacetic acid (TCA) followed by precipitation with alcohol and hydrolysis.

### Histopathological study

The histopathological studies of liver and pancreas were carried out at the Department of Pathology, Rajshahi Medical College, Rajshahi, Bangladesh. Briefly, for light microscopy liver and pancreas were fixed in PBS containing 10 % formalin. The tissues were washed in running tap water, dehydrated in the descending grades of isopropanol and finally cleared in xylene. The tissues were then embedded in molten paraffin wax. After embedding in paraffin, several transverse sections (5 μm) were cut from the mid organ level and stained with hematoxylin-eosin stain. The specimens were observed under light microscope at the 400-fold magnification.

### In vitro antioxidant activity of GAE extract by DPPH free radical scavenging assay

The antioxidant property was assessed by DPPH (2, 2-diphenyl-1-picrylhydrazyl) radical scavenging method [36]. The hydrogen donating or radical scavenging ability of the extract was measured using a stable radical DPPH. 2.8 ml of DPPH solution (45 μg/ml) were rapidly added in 200 μl of methanol solution of plant extracts at different concentrations in test tubes. The solutions were mixed well and then kept in dark for 30 min at room temperature. The absorbance was measured at 517 nm in spectrophotometer against methanol solution used as a blank. Ascorbic acid was used as standard and trolox in the same concentrations was used as the positive control corresponding to 100 % radical scavenging activity. All measurements were done in triplicate.

The percentage (%) of scavenging of the DPPH free radical was measured by using the following equation:

$$ \left\{\left({\mathrm{A}}_0-{\mathrm{A}}_1\right)/{\mathrm{A}}_0\right\}\times 100 $$

Where, A_0_ = absorbance of the control

A_1_ = absorbance of the extract/standard

Then, the percentage (%) of inhibition was plotted against log concentration and IC_50_ was calculated from the graph.

### Determination of total phenolic content in GAE

Total phenol content in extract was determined by Folin-Ciocalteu reagent [37]. Briefly, the extract (200 μg/ml) was mixed with 400 μl of the Folin-Ciocalteu reagent and 1.5 ml of 20 % sodium carbonate. The mixture was shaken thoroughly and made up to 10 ml with distilled water and after 2 h absorbance of the mixture was measured at 765 nm. The total phenol content in GAE extract was determined from standard curve of gallic acid and was expressed as mg of gallic acid equivalent per gm of dried plant extract.

### Determination of total flavonoid content in GAE

The total flavonoid content was determined using a method previously described by Kumaran K [38]. In brief, 1 ml of plant extract in ethanol (200 μg/ml) was mixed with 1 ml aluminium trichloride in ethanol (20 mg/ml), a drop of acetic acid was added and then diluted with ethanol up to 25 ml. After 45 mins absorbance was measured at 415 nm against blank. The total flavonoid content in GAE extract was determined from the standard quercetin curve and was expressed as mg of quercetin equivalent per gm of dried plant extract.

### Statistical analysis

Data were expressed as means ± standard error of means (SEM). Statistical comparison was performed by one-way (ANOVA) followed by Dunnett’s Multiple Comparison Test. The values were considered as statistically significant when *p* <0.05. Statistical calculations and the graph were prepared using GraphPad Prism Software version 5.0 (GraphPad Software, San Diego, CA, USA).

## Results

### Acute toxicity study

The oral administration of GAE in rats up to the dose 2000 mg/kg neither exhibit any sign of toxicity nor any rat died during the 14 days period. It indicates that GAE was nontoxic in rats up to the oral dose of 2000 mg/kg body weight. We therefore, carried out our investigations with 1/5^th^ and 1/10^th^ dose of GAE that is 400 and 200 mg/kg dose levels.

### Clinical course

Table 1 shows the survival rate among the group of rats after 15 days of treatment. Three (60 %) of five rats in Group DC died between days 7 to 15. None of the rats died in groups GAE200, GAE400 and DS. The 15 days survival rate was significantly higher among the treatment groups than in Group DC (*p* <0.01).

**Table 1:** Survival rate of rats after 15 days of oral treatment

Group (*n* = 5)	Total animal	Survivors	Deaths	Survival rate (%)
Normal control (NC)	5	5	0	100**
Diabetic control (DC)	5	2	3	40††
Diabetic standard (DS)	5	5	0	100**
GAE 200 (200 mg/kg)	5	5	0	100**
GAE 400 (400 mg/kg)	5	5	0	100**

Data expressed in percentages (%). Control group received 0.5 % methyl cellulose, standard group received 150 mg/kg Metformin, GAE200 and GAE400 received 200 and 400 mg/kg *Grewia asiatica* bark extracts. ††*p* <0.01 compared to NC, **p <0.01 compared to DC

### Effect of GAE on OGTT

After oral ingestion of glucose, the blood glucose levels were significantly higher among the diabetic rats as shown in Table 2. In Group DC, blood glucose concentration were peaked after 30 min and remained high over the next 90 min. Rats in Group GAE200 and GAE400 showed a significant attenuation in blood glucose concentration at 60, 90 and 120 min as compared with Group DC rats. However, the GAE400 showed greater improvement of glucose tolerance in diabetic rats and was comparable to that of Group DS (*p* <0.001).

**Table 2:** Effects of GAE on oral glucose tolerance test

Group (*n* = 5)	Time				
	0 min	30 min	60 min	90 min	120 min
NC	4.8 ± 1.0	8.9 ± 2	6.8 ± 2	6.1 ± 0.8	5.7 ± 1
DC	13.9 ± 1.3†††	18.5 ± 3.5†	15.8 ± 1†††	15.2 ± 1.4†††	14.9 ± 3†††
DS	10.9 ± 1.1†	11.9 ± 1.2	9.8 ± 4*	5.9 ± 0.7***	5.9 ± 3***
GAE200	12.8 ± 1††	16.9 ± 1.7†	10.1 ± 1*	9.1 ± 0.8**	7.5 ± 2.1**
GAE400	13.6 ± 1.4†††	19 ± 2.0†	12.3 ± 2*	7.3 ± 0.7***	7.1 ± 2.5***

Data expressed as means ± SEM. † *p* <0.05, †† *p* < 0.01, ††† *p* < 0.001 compared to NC, *p <0.05, **p <0.01, *** *p* < 0.001 compared to DC

### Time course of changes in blood glucose levels

**Fig. 1:**
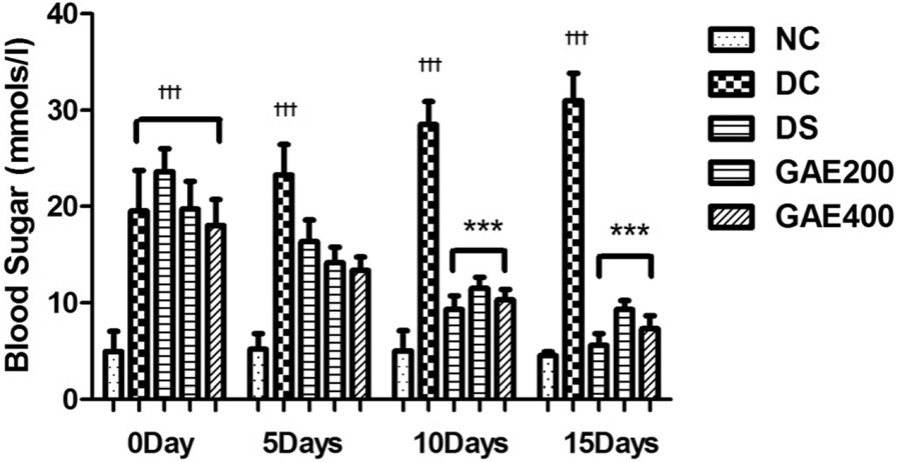
Time course of changes in blood sugar levels after oral administration of GAE in diabetic rats. Data expressed as means ± SEM. Each group comprised of five animals. NC and DC rats received 0.5 % MC and DS rats received 150 mg/kg Metformin, GAE200 and GAE400 received 200 and 400 mg/kg *Grewia asiatica* bark extracts. †*p* <0.05, ††*p* <0.01, †††*p* <0.001 compared to NC, *p <0.05, **p <0.01 and ***p <0.001 compared to DC

Time course of changes in blood sugar levels are shown in Fig. 1. On day 0, before the initiation of treatment the blood glucose levels were significantly higher in DC rats compared to NC rats (*p* <0.01). Oral administration of GAE significantly lowered the blood glucose levels on day 5, 10 and 15, and the effect was dose-dependent. During the course of treatment no significant differences in blood glucose levels were observed among the treatment groups.

### Effect of GAE on body weight and organ weight changes

Body weight, organ weights and organ weight to body weight ratios are summarized in Table 3. After 15 days of oral administration of GAE, body weights and organ weights were decreased in Group DC as compared to Group NC rats. Although treatment with GAE improved body weights, organ weights and organ weight to body weight ratios, the effects were not significant among the treatment groups.

**Table 3:** Changes on body weight and organ weight after oral administration of GAE in diabetic rats

Group (*n* = 5)	Body weight (g)	Heart weight (g)	Liver weight (g)	Pancreas weight (g)	H/B (g/kg)	L/B g/kg)	P/B (g/kg)
NC	150 ± 7	0.48 ± 0.025	5.51 ± 0.47	0.40 ± 0.04	3.1 ± 0.1	35.5 ± 1.23	2.3 ± 0.4
DC	120 ± 6†	0.34 ± 0.025†	3.5 ± 0.22†	0.25 ± 0.02†	2.6 ± 0.2	26.5 ± 2.7†	2.0 ± 0.08
DS	143 ± 7	0.49 ± 0.03	5.1 ± 0.81	0.38 ± 0.04	3.2 ± 0.22	34.2 ± 2.5	2.2 ± 0.26
GAE200	136 ± 6	0.47 ± 0.035	4.23 ± 0.26	0.30 ± 0.03	3.25 ± 0.1	30 ± 1	2.1 ± 0.1
GAE400	130 ± 5	0.45 ± 0.023	4.11 ± 0.25	0.26 ± 0.035	3.3 ± 0.23	30.5 ± 1.1	2.0 ± 0.02

Data expressed as means ± SEM. *H/B* ratio of heart weight to body weight, *L/B* ratio of liver weight to body weight, *P/B* ratio of pancreas weight to body weight. †*p* <0.01 compared to NC

### Effects of GAE extract on lipid profile

Table 4 represents the changes in lipid profile in diabetic rats. The data revealed that both TC and TG levels were significantly elevated in Group DC rats. The TC and TG levels were significantly reduced in Group GAE200 and GAE400 when compared with Group NC rats. The plasma HDL level was significantly lower and the levels of VLDL, LDL and LDL/HDL ratio were significantly higher in Group DC. Administration of GAE significantly restored the level of VLDL, LDL, HDL and the LDL/HDL ratio. The effects of GAE on lipid profiles were dose dependent. Among the extract treated rats, GAE400 exhibited greater improvement in lipid profile.

**Table 4:** Changes on lipid profile after oral administration of GAE in diabetic rats

Group (*n* = 5)	Total cholesterol (mg/dl)	Triglycerides (mg/dl)	VLDL (mg/dl)	LDL (mg/dl)	HDL (mg/dl)	LDL/HDL
NC	70.0 ± 7.4	71.7 ± 6.6†	14.33 ± 4.5	37.6 ± 5.6	29.0 ± 5.7	1.3 ± 0.37
DC	136.7 ± 14.7†††	115.7 ± 5.88†††	23.13 ± 1.1†††	76.3 ± 4.32†††	5.7 ± 1.77†††	13.2 ± 0.6†††
DS	79.0 ± 6.27**	73.5 ± 6.16***	9.8 ± 6.5***	25.0 ± 5.56***	41.5 ± 2.12***	0.60 ± 0.06***
GAE200	68.3 ± 5.65***	83.7 ± 5.01**	16.7 ± 1**	18.0 ± 3.74†***	29.3 ± 3.62***	0.61 ± 0.08***
GAE400	67.3 ± 8.04***	77.0 ± 3.74***	15.4 ± .74**	16.3 ± 2.94†***	34.3 ± 2.01***	0.48 ± 0.05***

Data expressed as means ± SEM. † p<0.05, ††† *p*<0.001 compared to NC, ** *p* < 0.01, *** *p* < 0.001 compared to DC

### Effect of GAE extract on liver glycogen, SGOT and CK-MB levels

The liver glycogen content, SGOT and CK-MB levels in diabetic rats were shown in Table 5. A significant decrease in the liver glycogen content was observed in Group DC rats. The values were significantly restored among the treatment groups whereby Group GAE400 exerted the most prominent improvement (*p* <0.05). After 15 days of oral ingestion of GAE, the increased level of SGOT and CK-MB were significantly attenuated in DC rats and was comparable to that of the NC rats.

**Table 5:** Changes on liver glycogen, SGOT and CK-MB levels after administration of GAE in diabetic rats

Group (*n* = 5)	Glycogen (g/100 g)	SGOT (U/l)	CK-MB (U/l)
NC	4.60 ± 0.31	8.33 ± 2.48	210 ± 14
DC	1.18 ± 0.57†††	25.0 ± 4.93††	378 ± 20†††
DS	3.95 ± 0.22***	9.0 ± 2.30**	185 ± 9***
GAE200	3.42 ± 0.25**	12.0 ± 2.24*	167 ± 11***
GAE400	4.23 ± 0.24***	7.0 ± 1.7**	152 ± 14***

Data expressed as means ± SEM. ††*p* <0.01, †††*p* <0.001 compared to NC, *p <0.05, **p <0.01, ***p <0.001 compared to DC

### Effect of GAE on DPPH free radical scavenging activity

**Fig. 2:**
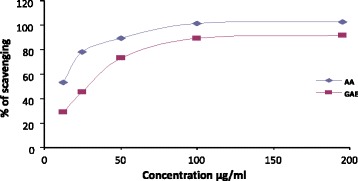
DPPH free radical scavenging activity (%) at various concentrations (μg/ml) of GAE bark extracts and ascorbic acid standard. The antioxidant activity of the extract was determined by the IC_50_ value

The antioxidant activity of GAE was assessed by in vitro DPPH free radical scavenging assay. The capability of GAE on reducing production of DPPH radicals at all concentrations are shown in Fig. 2. Ascorbic acid, used as standard was highly effective in inhibiting the DPPH free radicals, showing an IC_50_, 12.50 μg/ml where as GAE exhibited IC_50_, 76.45 μg/ml.

### Phytochemical screening

Phytochemical analysis of the crude extract of *G. asiatica* revealed the presence of flavonoid, steroid, glycoside, saponin, tannin, and triterpinoid (Table 6).

**Table 6:** Phytochemicals of *Grewia asiatica* stem bark

Extract	Alkaloid	Glycoside	Tannin	Triterpene	Saponin	Flavonoid
*GAE*	−	+	+	+	+	+

Sign (+) indicates present and sign (−) indicates absent

### Determination of total phenolic and total flavanoid content

The total phenol and total flavanoid content are shown in Table 7. As determined by the spectrophotometer, the total phenolic content of the extract was 44.65 ± 3.17 mg gallic acid equivalents/gm of GAE extract and the total flavonoid content was 39.11 ± 4.65 mg of quercetin equivalent/gm of GAE extract.

**Table 7:** Total phenol and total flavonoid content in *Grewia asiatica* stem bark

Scientific name	Total phenol content (mg of gallic acid equivalent /gm of dried extract)	Total flavonoid content (mg of quercetin /gm of dried plant extract)
*G. asiatica*	44.65 ± 3.17	39.11 ± 4.65

### Histopathological investigation

**Fig. 3:**
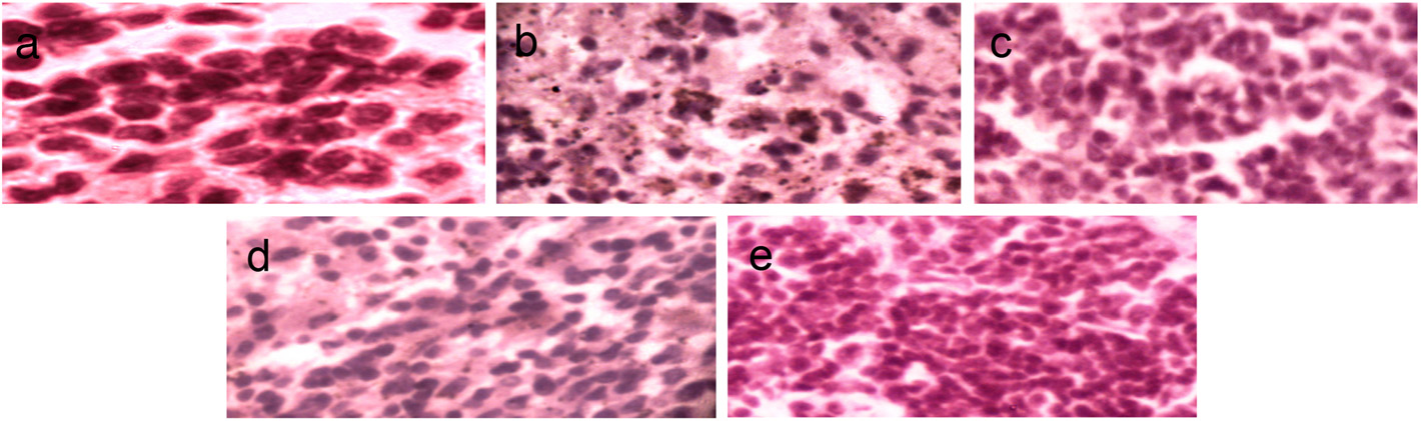
Hematoxylin-eosin staining of the cross sectional areas of pancreas. Original magnifications 400×. **a** NC rats showing intact architecture and normal cellular population of pancreatic β-cells. **b** DC rats indicating inflammation and damaged islets. **c** DS rats showing partial restoration of pancreatic β-cells and absence of islets damage. **d** GAE 200 showing mild to moderate restoration normal cellular population size of pancreatic β-cells and absence of islets damage (**e**) GAE 400 showing maintenance of normal architecture of pancreatic β-cells with hyperplasia

Figure 3 illustrates representative photographs of thin sections of pancreas stained with Hematoxylin-eosin, where NC rats showed normal cellular population in the islets of Langerhans in pancreas. The islets of diabetic rats showed extensive damage and inflammations with loss of normal architecture of pancreatic β-cells. The rats treated with GAE showed significant improvement in cellular architecture as observed by the restoration of normal cellular population size of islets with hyperplasia.

## Discussion

Diabetes is multifactorial disease that has a significant adverse impact on health and mortality particularly from cardiovascular diseases. Now, a day, herbal drugs are gaining popularity in the treatment of diabetes and its related complications. The present study was designed to assess the hypoglycemic, hypolipidemic and antioxidant activities of ethanolic extract *of G. asiatica* stem barks in alloxan-induced diabetic rats for 15 days. Alloxan is a hydrophilic and chemically unstable pyrimidine derivative which can generate free radicals that are toxic to pancreatic β-cells causing rapid release of insulin initially and then sharp decline due to excess liberation of stored insulin and in this study, the diabetogenic effect of alloxan was in accord with previous studies [39]. Beside insulin, the most widely used hypoglycemic agents are sulfonylureas and biguanides. However, we choose metformin- a biguanides as a standard drug which inhibit gluconeogenesis in the liver, increases affinity to insulin receptors and thus, improve insulin resistance [40].

The present study indicated that 15 days of oral administration of GAE improved survival rate and significant reduction in blood glucose, lipids, SGOT, CKMB levels and restored liver glycogen in diabetic rats. After 15 days diabetic rats showed a significant improvement in glucose tolerance and the effects of GAE on blood sugar levels and biochemical alterations were dose-dependent. Remarkably, the rats treated with GAE showed mild to moderate improvement in cellular architecture as observed by the restoration of normal cellular population size of islets.

We demonstrated that GAE at the doses of 200 and 400 mg/kg reduced elevated blood sugar level in alloxan-induced diabetic rats. Our results were in accordance with outcomes of Parveen et al. showed antihyperglycemic activity of different parts of *G. asiatica* in alloxan-induced rabbits [25]. A number of medicinal plants have been reported to have an antihyperglycemic activity and a stimulatory effect on insulin release [41, 42]. The significant decrease in the fasting blood glucose levels by GAE in alloxan diabetic rats may be due to the stimulation of the residual pancreatic mechanism and probably by increasing peripheral utilization of glucose or glycogen synthesis in liver and decreased gluconeogenesis [43].

Induction of diabetes with alloxan is associated with characteristic loss of body and organ weight, which is due to increase muscle wasting [44] and loss of tissue proteins [45]. Diabetic rats treated with GAE showed an increase in body weight and organ weight which may be due to protective effect of GAE on tissue structural constituents [46].

Hyperglycemia is accompanied with the increase in TC, TG, LDL and decrease in HDL which is attributable to excess mobilization of fat from the adipose due to under peripheral utilization of glucose [47]. The data revealed that TC, TG, LDL, VLDL levels were significantly decreased and HDL level increased in diabetic rats treated with GAE. The Group GAE400 exhibited greater improvement in lipid profile among the treatment groups. Oral administration of GAE might have improved utilization of glucose and suppression of lipid mobilizations responsible for the regression of diabetic state. Further, the effects may be due to the low activity of cholesterol biosynthesis enzymes and/or low level of lipolysis which is under the control of insulin [48]. It is evident that triglycerides are independent risks factors of coronary heart diseases [49] and most of the lipid lowering drug does not decrease TG levels. However, GAE lowered TG levels significantly and this effect might be due to an increase in endothelium bound lipoprotein lipase which regulates the disposal of lipids fuels in the body [50].

The SGOT and CK-MB are sensitive markers of organ damage [51]. The levels of SGOT and CK-MB were abnormally increased alloxan induced diabetic rats. The increase in SGOT levels might be due to hepatotoxicity and however, the cause of high levels CK-MB remained to be explained. Oral ingestion of GAE significantly reduced SGOT and CK-MB levels among the treatment groups suggestive of improvement in liver function and morphology in diabetic rats (Table 5). In our study, induction of diabetes with alloxan was associated with a marked reduction in liver glycogen stores which could be attributed to a decrease in the availability of the active form of enzyme glycogen synthetase probably because of low level of insulin [52]. Oral administration of GAE restored the liver glycogen content possibly due to an increase level of insulin, which was evident by the preservation of pancreatic morphology and regeneration of β-cells (Fig. 3d and e). *Vinca rosea* extracts and (−)-Epicatechin have been shown to induce β-cell regeneration in alloxan-induced diabetic rats [53, 54]. In our studies, the damaged pancreatic β-cells were observed in diabetic rats (Fig. 3b). However, oral ingestion of GAE restored normal population size of islets by the regeneration of β-cells (Fig. 3d and e). The antioxidant activity of the plant extract might play a significant role in the early recovery of damaged pancreas in diabetic rats which in turn may be due to the presence of flavonoid and phenolic compounds in *G. asiatica* stem bark.

## Conclusion

We concluded that ethanol extract of *G. asiatica* stem barks has antidiabetic and lipid lowering efficacy in alloxan-induced diabetic rats. The plant extract exerts its beneficial effects by the reduction of blood sugar levels, lipid profiles, SGOT and restoration of liver glycogen and antioxidant potentials. GAE treated pancreas showed maintenance of normal architecture of pancreatic β-cells as evidenced in histological findings. Thus, the antihyperglycemic effects of GAE can be partially explained by their ability to restore the functions of pancreatic tissues. However, further histopathological and biochemical studies are needed to elucidate the exact mechanism of action of *G. asiatica* in diabetic rats.

## Abbreviations

DM: Diabetes Mellitus
GAE: *Grewia asiatica Extract*
ICDDRB: International Centre for Diarrhoeal Disease Research, Bangladesh
SGOT: Serum Glutamate Oxaloacetate Transaminase
CK-MB: Creatine Phosphokinase MB Isoenzyme
DPPH: 2, 2-diphenyl-1- picrylhydrazyl
TC: Total Cholesterol
TG: Triglycerides
VLDL: Very Low Density Lipoprotein Cholesterol
LDL: Low Density Lipoprotein Cholesterol
HDL: High Density Lipoprotein Cholesterol
